# Cytology of the vulva: feasibility and preliminary results of a new brush

**DOI:** 10.1038/bjc.2011.533

**Published:** 2011-12-01

**Authors:** L C G van den Einden, J M M Grefte, I A M van der Avoort, J E M Vedder, L C L T van Kempen, L F A G Massuger, J A de Hullu

**Affiliations:** 1 Department of Obstetrics and Gynaecology, Radboud University Nijmegen Medical Centre, Nijmegen 9101, The Netherlands; 2 Department of Pathology, Radboud University Nijmegen Medical Centre, Nijmegen, The Netherlands

**Keywords:** vulvar intraepithelial neoplasia, vulvar brush, vulvar cytology, vulvar dysplasia

## Abstract

**Objective:**

Taking a biopsy is a standard procedure to make the correct diagnosis in patients with suspicious premalignant vulvar lesions. The use of a less invasive diagnostic tool as triage instrument to determine whether biopsy is necessary may improve patient comfort especially in patients with chronic vulvar disorders that may warrant consecutive biopsies. This study was conducted to investigate whether vulvar brush cytology is feasible and may be used to detect (pre)malignant vulvar lesions.

**Methods:**

A pilot study was performed with patients having clinically normal vulvar skin, lichen sclerosus (LS), usual or differentiated vulvar intraepithelial neoplasia or squamous cell carcinoma. A total of 65 smears were taken with the use of a vulvar brush and biopsies were performed for histopathological analysis.

**Results:**

Out of 65 smears, 17 (26%) were discarded because of poor cellularity. A total of 28 of 29 (97%) smears with a histological proven (pre)malignancy had a smear classified as ‘suspicious’ or ‘uncertain’. Cytology classified 11 smears as ‘non-suspicious’, of which 10 (91%) were indeed normal skin or LS. The accuracy, based on the presence of a lesion, for (pre)malignant lesions with the use of the brush showed a sensitivity of 97% and a negative predictive value of 88%.

**Conclusion:**

Vulvar brush cytology is feasible and may be a first step in the development of a triage instrument to determine whether subsequent biopsy of a clinically (pre)malignant lesion is necessary.

Vulvar squamous cell carcinoma (SCC) is a multifactorial disease following two separate and independent pathways. Each pathway has its own precursor lesion (Maclean, 2006; van der Avoort *et al*, 2006); usual VIN (uVIN) is the first precursor and is caused by the human papilloma virus (HPV). Differentiated vulvar intraepithelial neoplasia (dVIN) is the second and most common precursor and often occurs in a background of lichen sclerosus (LS) (Yang and Hart, 2000; Eva *et al*, 2009). On the basis of our earlier studies we conclude that dVIN and not LS is the true precursor of SCC (van de Nieuwenhof *et al*, 2010).

Patients with LS have a lifetime risk of 4–6% to develop vulvar SCC (Meffert *et al*, 1995; Hsieh and Kuo, 2004). Therefore, life-long follow-up is advised. In case of suspicion of a vulvar (pre)malignancy, histopathological examination is required and considered to be the gold standard. Usually punch biopsies are conducted under local or general anaesthesia (van de Nieuwenhof *et al*, 2008). After treating vulvar (pre)malignancies there is often residual disease and/or a high risk of recurrent lesions. In these patients, the vulvar examination can be difficult because of scarring due to a previous vulvectomy. Besides, the majority of these patients fear repeated biopsies making the development of a less invasive, accurate, diagnostic tool desirable to improve patient comfort. Brush cytology has been proven to be a reliable patient-friendly method to diagnose cervical (pre)malignancies. The accuracy of cytology largely depends on the presence of enough cells and the ability to recognise cellular and nuclear atypia. Various techniques for vulvar cytology have been described with disappointing results because of scarce cellularity so vulvar cytology is now far from being common practice (Dennerstein, 1968; Nauth and Boger, 1982a; Nauth and Schilke, 1982b; Kashimura *et al*, 1993; Levine *et al*, 2001; Jimenez-Ayala and Jimenez-Ayala, 2002; Bae-Jump *et al*, 2007).

For the present pilot study, a new vulva brush (Rovers Medical Devices BV, Oss, The Netherlands; Figure 1) for obtaining vulvar cytology was introduced for a feasibility study at our department. A non-invasive tool was designed, resembling the cervex-brush, but with a brushing surface suitable for the vulvar skin and the ability to collect enough cells for cytology. Though histology still remains the gold standard, with this brush we want to make a first step in the development of a triage instrument that can determine whether subsequent biopsy of a clinically (pre)malignant lesions is necessary. This study was conducted to investigate whether vulvar cytology obtained by this brush is feasible and may be used in distinguishing benign from (pre)malignant vulvar lesions.

## Patients and methods

The pilot study was performed in patients from the vulvar clinic of the Department of Obstetrics and Gynaecology at the Radboud University Nijmegen Medical Centre, The Netherlands. Over a period of several months, 37 women were recruited having clinically LS, lesions suspicious of uVIN (raised, well-demarcated and asymmetrical lesions; varying from white and condylomatous-like to brown lesions) or dVIN (raised white plaque, ulcerative or erythematous red lesion) or SCC (ulcerative lesions). All patients underwent cytological brushing and one or more vulvar punch or excisional biopsies were performed. A biopsy was not performed at the site of the brushing of the normal skin.

Brushing was performed only at the site of the lesion; in patients with LS the most affected site was chosen to brush. Saline moistening was used before brushing, in order to remove debris, ointment and/or keratinised squamous cells as much as possible. All smears were prepared following our local standard Thin Prep protocol, using the Thin Prep 3000 Processor (Cytyc Europe Benelux, Almere, The Netherlands), Papanicolaou stained and subsequently (blindly) assessed by both an experienced cytotechnologist (JV) and an expert cytopathologist (JG).

All smears were evaluated and scored for cellularity, presence of hyper and parakeratosis, presence of koilocytosis, atypia and squamous cell dysplasia. Regarding cellularity, a slight modification of the Bethesda 2001 guidelines for cervical cytology was followed (Solomon *et al.*, 2002). In short, <5000 squamous cells and/or anucleate squamous cells per slide were considered inadequate, >5000 but <8000 were suboptimal and >8000 were considered sufficient. If cellularity was adequate, smears were classified as ‘suspicious for (pre)malignancy’, ‘uncertain’ or ‘non-suspicious’. Also the most likely corresponding histological disorder (uVIN or dVIN) was scored based on the cytological findings present in the slide (Table 1).

**Figure 2:**
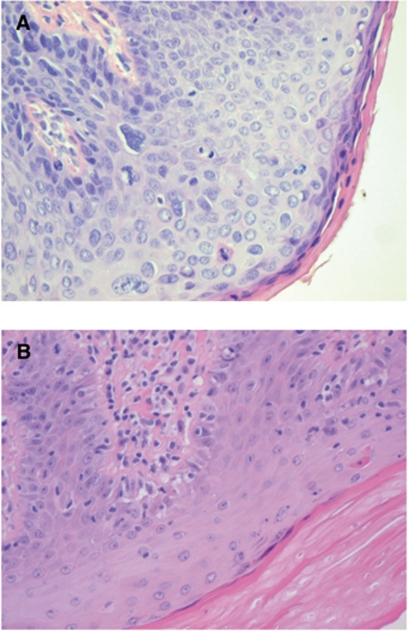
Histology of uVIN and dVIN. (**A**) uVIN; atypia and mitoses are present in all levels of the epidermis, nucleo–cytoplasmic (N/C) ratio is increased and koilocytes can be seen. (**B**) dVIN; normal N/C ratio, atypia confined to the (para)basal layers of the epithelium, the superficial layer shows normal maturation with a single dyskeratotic cell and prominent hyperkeratosis. No koilocytosis (H&E-stained, × 20).

**Figure 3:**
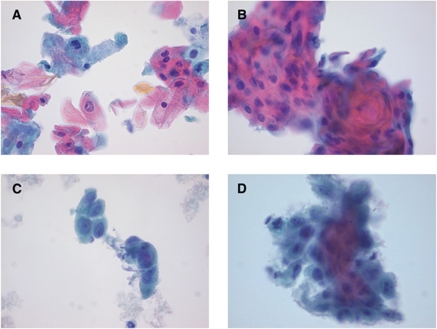
Cytological findings consistent with uVIN and dVIN. (**A** and **B**) uVIN: dyskeratotic cells and cell groups with increased N/C ratio, irregular coarse chromatin and irregular nuclear membranes. (**C** and **D**) dVIN: presence of large atypical cells with eccentric nuclei with prominent nucleoli and relatively abundant cytoplasm (Papanicolaou stained thin prep samples, × 40).

Histological features of uVIN are well recognisable. Atypical cells with increased nucleo-cytoplasmic (N/C) ratio are present at all levels of the epidermis and koilocytes may be numerous. In addition, the chromatin pattern is coarse and mitoses may be numerous (Figure 2A). Histological features of dVIN are more difficult to recognise. Atypia is confined to the (para)basal layers of the epithelium, in which the cells have abundant cytoplasm and may form abortive pearls. Prominent nucleoli are often present. Mitoses may be frequent, but are confined to the (para)basal cell layers. The superficial layers of the epithelium show a normal maturation and do not contain koilocytes. However, individual dyskeratotic cells may be seen. Furthermore, a thick hyper and sometimes parakeratotic layer is often present (Figure 2B). Invasive carcinoma will show a disrupted basement membrane with infiltrating nests of atypical squamous cells surrounded by a desmoplastic stroma reaction. Therefore, the following criteria for cytology were scored: if koilocytes, dyskeratotic squamous cells and cells with increased N/C ratio were present, smears were categorised as suspicious for (pre)malignancy, favour uVIN (Figure 3A and B): if large atypical epithelial cells with prominent nucleoli, eccentric nuclei and abundant non-keratinising cytoplasm were present, in the absence of the above described characteristics, smears were categorised as suspicious for (pre)malignancy, favour dVIN (Figure 3C and D). If only a few atypical cells were present, or if cells showed only slight aberrations, smears were categorised as ‘uncertain’. No attempt was made to specifically diagnose invasive SCC, as this cannot be differentiated reliably from the precursor lesions dVIN and uVIN on cytological brush material.

All biopsies and excision specimens were routinely fixed (4% buffered formalin) and paraffin embedded. Standard 4-*μ*m thick haematoxylin and eosin-stained sections were used for the classification of the lesions according to current WHO criteria and the recent modification of the ISSVD (Sideri *et al*, 2005). Finally, cytological and histological findings were correlated. The study was conducted after obtaining local ethics committee approval from the Radboud University Nijmegen Medical Centre, Nijmegen, The Netherlands, and informed consent of all participants.

### Statistics

Calculations were performed using SPSS 16.0 (SPSS Inc., Chicago, IL, USA). Descriptive statistics were used to reproduce study results as percentages, means and medians.

## Results

A total of 65 smears with the vulva brush were taken from 37 patients; in 40 of 65 smears a vulvar punch or excisional biopsy was taken immediately adjacent to the brushed area and in 13 of 65 smears a biopsy was taken a median of 5 months before or after the smear. Additionally, in 12 of 37 patients a smear was taken of clinically normal vulvar skin far from the lesion. Brushing was feasible in the outpatient clinic and well tolerated by the patients.

A total of 17 smears (26%) were inadequate because of poor cellularity and excluded from further analysis. Of these excluded smears the diagnosis was LS in 7 of 17 smears (histologically confirmed; 41%) and clinically normal in 7 of 17 smears (41%); an overview of the exact diagnoses can be seen from Table 2. Besides, 18 smears (28%) had a suboptimal cellularity and 30 (46%) had sufficient cellularity.

The correlation between the cytological and histological diagnoses is shown in Table 2. Among the 48 smears when suboptimal or sufficient cellular smears were obtained, 29 were biopsy proven (pre)malignancies; uVIN (*n*=14), dVIN (*n*=3) or vulvar carcinoma (*n*=12). A total number of 28 of 29 (97%) biopsy proven (pre)malignancies had a smear classified as ‘uncertain’ or ‘suspicious for (pre)malignancy’. Only one biopsy proven case of dVIN had a corresponding smear classified as ‘non-suspicious’. However, the cellularity of this one false-negative smear was suboptimal and predominantly anucleated squamous cells were present.

Diagnosis of 19 of the remaining 48 smears were biopsy-proven LS (*n*=14) or samples of normal skin (*n*=5). In 10 of these 19 cases (53%) the corresponding smear was correctly classified as ‘non-suspicious’. Seven smears were classified ‘uncertain’, of which six were histologically diagnosed as LS. Two smears were ‘suspicious for (pre)malignancy’ and were diagnosed as LS and normal.

Although we are aware of the effect the small sample size, we calculated the accuracy of cytology to diagnose a malignancy and/or premalignancy based on the presence of a lesion. Smears of normal skin were excluded for this calculation as it was our purpose to use vulvar cytology to distinguish between benign and (pre)malignant vulvar lesions. Accuracy is shown in Table 3. Cytology has a 100% sensitivity and negative predictive value of 100% in case of a malignancy. In case of premalignancies (uVIN and dVIN), sensitivity of 94% and a negative predictive value of 88% was obtained. For malignant and premalignant samples together 97% sensitivity and a negative predictive value of 88% was calculated. Specificity was 50% for both premalignancy and malignancy. The accuracy of only the smears taken immediately adjacent to the place of biopsy (*n*=52) showed comparable results.

## Discussion

Obtaining a rapid and accurate diagnosis in women suspected of VIN or vulvar cancer generally leads to (repeated) punch biopsies and consequent patient discomfort. Though histology remains important as it is currently the gold standard, especially for the primary diagnosis of LS, our results indicate that cytology obtained by the new vulva brush is feasible. Moreover, it may be a possible first step in the development of a triaging instrument to determine which patients with the suspicion of a pre(malignancy) especially in the follow-up, should have a subsequent punch biopsy and in which patients a biopsy might be safely omitted.

In this study, 17 of 53 smears (26%) did not carry enough cells for interpretation. This difficulty in obtaining sufficient material has also been discussed in the previous literature (Nauth and Boger, 1982a; Nauth *et al*, 1987; Dennerstein, 1988; Jimenez-Ayala and Jimenez-Ayala, 2002; Bae-Jump *et al*, 2007). Whether our brushing technique results in higher cellularity compared with other techniques with a spatula or blade is not clear because studies with different techniques do not use comparable analysing methods. Moreover, the cellularity in vulvar smears is much lower compared with cervical smears. This can be explained by the presence of a thick keratin layer that covers the vulvar epithelium, which requires vigorous brushing to obtain the underlying diagnostic cells for adequate sampling. Additionally, debris and/or keratinous squamous cells may confuse the cytological appearance of the sample; in our study we tried to prevent this by cleaning the surface with saline before brushing. Low cellularity may also be explained by the type of lesion that was brushed; when looking more in detail to the diagnoses of the 17 smears with poor cellularity (Table 2), it is striking and reassuring that the histological findings of these smears are benign. The small proportion of (pre)malignancies with low cellularity (*n*=3), supports the hypothesis that (pre)malignant lesions may dissociate more easily compared with benign lesions. In this study, brushing was not performed according to a strict protocol, which may have led to a variation in cell collection. For optimising the brushing method a more standardised approach should be followed. This approach should first contain repeatedly firm brushing of the surface. In some patients this may be painful. Probably the use of local anaesthetics such as Xylocaine spray can be helpful in these cases. Second, in cases with remaining low cellularity the use of immunohistochemistry should be considered. There might be an important role for HPV testing and the use of markers such as p53 or p16 to make a distinction between benign, HPV-related (usual) VIN/SCC or HPV-non-related (differentiated) VIN/SCC (Van der Avoort *et al*, 2006). Recently, an attempt was made to identify a new marker of specifically dVIN. van de Nieuwenhof *et al* (2010) showed localisation of mast cells in dVIN, which could be a potential marker for this entity. We hypothesise that the use of markers may lead to adequately differentiation, also in the samples with poor cellularity. Whether this marker will be useful in liquid-based cytology, can be subject of future studies.

Only three prior studies with a limited number of samples, have evaluated whether results from cytology correlate with histological findings of VIN or SCC. Bae-Jump *et al* (2007) concluded that with the use of a spatula end for cytology collection, only 7 of 22 patients (32%) with biopsy proven (pre)malignancies had a vulvar Pap smear significant for VIN or vulvar carcinoma. They concluded that a negative Pap smear was not necessarily indicative for the absence of disease. Likewise, we found one smear with normal cytology, but with a histologically proven dVIN. This cytological misdiagnosis might be explained by suboptimal cellularity and the presence of predominantly anucleated squamous cells in the preparation. As it is known that the atypical cells in dVIN reside predominantly in the (para)basal cell layers, probably in this case brushing was not performed vigorously enough to obtain diagnostic atypical cells. Furthermore, vulvar brush material often contains many anucleated squamous cells. Therefore, in the future a cytological specimen should not only be analysed for cellularity before making a diagnosis, but also additionally be assessed for the presence of enough nucleated squamous cells.

Jimenez-Ayala and Jimenez-Ayala (2002) collected cells for cytology (*n*=563) by scraping with a scalpel blade. They reported that cytology can be used for the diagnosis of malignancy with a sensitivity of 98%, which is comparable to our results, although they also reported a high specificity of 95%. Their better results compared with ours may be due to a more vigorous scraping method in which they probably collected more cells from the deeper tissue layers, underscoring the remarks above. However, patient discomfort was not scored and the accuracy of detecting premalignancies was not investigated in the study.

The accuracy obtained with our vulvar brush is different from the accuracy that can be obtained with the cervical brush to diagnose cervical (pre)malignancies. The accuracy of routine cytology is highly variable in different studies. An overview of Cuzick *et al* (2006) showed that the overall sensitivity (53%, range 18.6–76.7%) is lower compared with the overall specificity (96.3%, range 84.2–99.6%) in detecting high-grade cervical intraepithelial neoplasia (2+). The primary aim of the coordinated screening programs for cervical cancer is early detection of (pre)malignancies of the cervix in healthy women. This aim is completely different from our study where brushing is used for patients with a suspicious lesion. In our study the sensitivity (97%) was higher compared with the specificity (50%), which is acceptable concerning the aim of triaging patients with suspicious vulvar lesions.

Levine *et al* (2001) used the cytobrush for cell collection and analysed 28 cytological samples of histologically benign or premalignant lesions. With dyskeratosis as the sole cytological criterion for VIN or anal intraepithelial neoplasia (AIN), all samples were consistent with histology except for one (4%) that was cytologically diagnosed as VIN, but no VIN was identified in the biopsy. Presumably, these were all HPV-related VIN or AIN cases; no attempt was made to differentiate between uVIN and dVIN. In contrast with Levine's percentage of false-positive smears (1 out of 28 smears; 4%), in our study 9 smears (19%) were classified as ‘uncertain’ (*n*=7) or ‘suspicious for (pre)malignancy’ (*n*=2). Cytological recognition of uVIN generally is easier because koilocytosis and dyskeratotic cells are present in the entire epithelium, whereas in dVIN atypical cells are only present in the basal layer. Because the aim was to diagnose uVIN and dVIN, the threshold of suspicion was lower with consequently more false positives in our study and therefore lower positive predictive value. However, compared with standard management of suspicious vulvar lesions, the amount of biopsies may decrease. We hypothesise that especially those patients, visiting the clinic on a regular basis with recurring premalignancies, may benefit from cytology as a triaging instrument. The experience in our vulvar clinic is in that patients with premalignancies such as uVIN, and in particular dVIN, lesions are difficult to distinguish from benign on the clinical findings. This implies that biopsies may be taken despite the absence of disease. Not seldom we performed a vulvar mapping where no (pre)malignant lesions can be found. In these patients we want to use cytology to safely exclude premalignancies. The next step is to perform a study in a larger group of patients to investigate whether cytology with our vulva brush indeed can function as a triage instrument.

This study shows that cytology obtained by the new vulva brush is promising as a possible first step in obtaining a diagnosis in patients with suspicious (pre)malignant vulvar lesions. By classifying the smear based on the presence or absence of a few parameters, it is possible to detect a (pre)malignancy. However, histology still remains the gold standard and the brushing technique needs to be improved and addressed in future studies to increase the cellularity, which is obligatory for the right diagnosis.

## Figures and Tables

**Figure 1:**
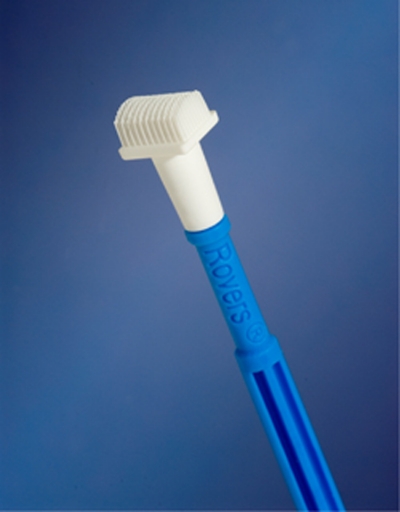
Vulva brush (Rovers Medical Devices BV).

**Table 1:** Classification of cytological smears

		**Suspicious for (pre)malignancy**	
**Normal**	**Uncertain**	**Favour uVIN**	**Favour dVIN**
No atypical or dysplastic cells	Some atypical cells	Evident dyskaryotic cells and cell groups Increased N/C ratio, irregular coarse chromatin, irregular nuclear membrane Koilocytes present	Large atypical cells, often isolated Eccentric nuclei Prominent nucleoli Absence of koilocytes

Abbreviations: dVIN=differentiated vulvar intraepithelial neoplasia; N/C ratio=nucleo-cytoplasmic ratio; uVIN=usual vulvar intraepithelial neoplasia.

**Table 2:** Cytology–histology correlation (*n*=65)

	**Histology**					
**Cytology**	**SCC**	**uVIN**	**dVIN**	**Lichen sclerosus**	**Normal skin** a	**Total**
*Suspicious for (pre)malignancy* b						
Favour uVIN	8	9	0	1	1	19
Favour dVIN	3	0	1	0	0	4
Uncertain^c^	1	5	1	6	1	14
Non suspicious	0	0	1	7	3	11
Poor cellularity	1	2	0	7	7	17
Total	13	16	3	21	12	65

Abbreviations: dVIN=differentiated vulvar intraepithelial neoplasia; SCC=squamous cell carcinoma; uVIN=usual vulvar intraepithelial neoplasia.

a Based on clinical appearance, no histologic confirmation.

b Atypical cells present; indicative of a (pre)malignancy.

c Presence of atypical cells not conclusive.

**Table 3:** Accuracy of vulvar cytology

**Diagnosis**	**Sensitivity (%)**	**Specificity (%)**	**Negative predictive value (%)**	**Positive predictive value (%)**
(Pre)malignancy^a^	97	50	88	80
Malignancy^b^	100	50	100	63
Premalignancy^c^	94	50	88	70

a Including usual vulvar intraepithelial neoplasia (uVIN), differentiated vulvar intraepithelial neoplasia (dVIN) and squamous cell carcinoma (SCC).

b Including SCC.

c Including uVIN and dVIN.
